# Reproducibility and intraindividual variation over days in buccal cell DNA methylation of two asthma genes, interferon γ (IFNγ) and inducible nitric oxide synthase (iNOS)

**DOI:** 10.1186/1868-7083-4-3

**Published:** 2012-02-01

**Authors:** DZ Torrone, JS Kuriakose, K Moors, H Jiang, MM Niedzwiecki, FF Perera, RL Miller

**Affiliations:** 1 Division of Pulmonary, Allergy and Critical Care of Medicine, PH8E, Columbia University Medical Center, 630 West 168th Street, New York, NY 10032, USA; 2 Department of Environmental Health Sciences, Mailman School of Public Health, Columbia University, 722 West 168th Street, New York, NY 10032, USA; 3 Department of Pediatrics, Columbia University Medical Center, PH8E, 630 West 168th Street, New York, NY 10032, USA

**Keywords:** methylation, asthma, IFNγ, iNOS, buccal mucosa, epigenetic regulation, pediatric, inner city

## Abstract

The biological mechanisms responsible for the onset and exacerbation of asthma symptoms in children may involve the epigenetic regulation of inflammatory genes after environmental exposures. Using buccal cells, we hypothesized that DNA methylation in promoter regions of two asthma genes, inducible nitric oxide synthase (iNOS) and interferon γ (IFNγ), can vary over several days. Repeat buccal samples were collected 4 to 7 days apart from 34 children participating in the Columbia Center for Children's Environmental Health (CCCEH) birth cohort study. Several field duplicates (sequential collection of two samples in the field) and replicates (one sample pyrosequenced twice) also were collected to ensure consistency with collection and laboratory procedures. DNA methylation was assessed by pyrosequencing a PCR of bisulfite-treated DNA. We found that replicate and field duplicate samples were correlated strongly (r = 0.86 to 0.99, *P *< 0.05), while repeat samples demonstrated low within-subject correlations (r = 0.19 to 0.56, *P *= 0.06 to 0.30). Our data reveal DNA methylation as a dynamic epigenetic mechanism that can be accessed safely and reproducibly in an inner city pediatric cohort using non-invasive buccal swabs and pyrosequencing technology.

## Introduction

The biological mechanisms responsible for the development of asthma symptoms in children following acute exposure to air pollution and other triggers are complex. These include the induction of oxidative stress pathways and formation of excessive reactive oxygen species in the airways [1-5]. Also, exposure to diesel and other combustion products may upregulate proallergic T helper (Th) 2 immune mechanisms [1,6-9]. Epigenetic regulation of gene expression associated with airway inflammation and allergic immune responses following exposure to air pollutants has been proposed as a key molecular step linking environmental exposures with altered asthma gene expression and asthma symptoms [10-14].

To date, clinical research on epigenetic changes in asthma and other complex diseases has been limited, especially in children [11,12]. One cross-sectional study by White and colleagues observed promoter demethylation of the allergy counter-regulatory and Th1 cytokine interferon γ (IFNγ) gene in association with *in vitro *differentiation of CD4+ neonatal T cells [15]. Another study by Kwon and colleagues found phytohemagglutinin (PHA) and dust mite allergen stimulation of CD4+ T lymphocytes induced small increases in the degree of demethylation in several CpG loci of the Th2 interleukin (IL)-4 promoter (CpG^-80^, CpG^+5^) in adult asthmatic subjects, when compared to the control group [16]. The changes in DNA methylation at the IFNγ promoter were less consistent. Recently, Breton and colleagues sampled children living in Southern California in one of the first large cohort studies analyzing DNA methylation of asthma genes in buccal cells [17]. They hypothesized that buccal cell DNA methylation levels in two genes important to the production of proinflammatory nitric oxide, namely arginase (ARG) and inducible nitric oxide synthases (iNOS), would be associated inversely with fractional exhaled nitric oxide (FeNO) levels measured concurrently at one timepoint. They found that methylation levels in the promoter regions of ARG1 and ARG2, but not iNOS, were associated inversely with FeNO levels among asthmatic children.

Despite these few advances, several fundamental questions still need to be elucidated in environmental epigenetic asthma research. Some relate to basic questions about quality assurance and controls, such as the reproducibility of biospecimen collected under 'real world' field conditions and their quantification of DNA methylation levels in the laboratory. Another is whether biologically relevant epigenetic marks change readily over time, even over the short term. Our objective was to answer such fundamental questions in a pediatric urban cohort using non-invasive duplicate and repeat sampling. Our approach was to collect duplicate and repeated buccal cells, collected as self-performed cheek swabs by children in the field (that is, the child's home), as an accessible population of aerodigestive tract cells that may undergo changes in gene expression following exposure to environmental toxicants (for example, environmental tobacco smoke (ETS)) in a manner that correlate with those derived from the airway [18-20]. Also, as described above, buccal cells demonstrate gene-specific DNA demethylation that has been associated with airway inflammation [17].

We also chose to measure DNA methylation of two representative asthma genes, namely IFNγ and iNOS. IFNγ is a well established negative regulator of airway allergic immune responses [21]. The induction of IFNγ primarily is regulated by demethylation of CpG sites within the IFNγ gene [5,15,22]. While Breton and colleagues did not find associations with iNOS demethylation in buccal DNA and FeNO production, Tarantini and colleagues found that fine particulate matter exposure over days was associated with iNOS demethylation in peripheral blood mononuclear cells (PBMCs) [23]. Our aims were (1) to determine the reproducibility of levels of DNA methylation at multiple CpG sites for both genes when collected as duplicate samples in the field, and (2) to determine whether changes in DNA methylation levels occur over days.

## Methods

### Collection of buccal cell DNA samples

Buccal samples were collected using the Cytosoft Cytology Brush in Qiagen's Puregene Buccal Cell Core Kit (Qiagen Sciences, Germantown, MD, USA) from 34 children aged 9 to 10 years old living in Northern Manhattan and the Bronx, NY, USA, participating in the Columbia Center for Children's Environmental Health birth cohort study [1,24,25]. Informed consent and assent were obtained from all participants prior to their participation in the study. Field technicians traveled to the subject's home and instructed children not to eat or drink for 1 h prior to cheek swab. Each child rinsed his/her mouth with water and then brushed the inside of his/her cheek for 1 minute. Upon completion, the field technician placed the swab immediately into 600 μl of cell lysis solution (Qiagen). For field duplicate samples, the children were given two swabs to brush inside their cheeks in immediate succession for 1 minute each.

### Extraction, quantification, and bisulfite conversion

Buccal cell DNA extractions were performed using Puregene Buccal Cell Core Kits (Qiagen) according to the manufacturer's instructions, except all centrifugations were conducted at 4°C instead of room temperature. Buccal DNA was quantified using PicoGreen (Invitrogen Corporation, Carlsbad, CA, USA) ultrasensitive fluorescent nucleic acid stain for double-stranded DNA. Bisulfite conversion was performed on 200 ng of genomic buccal cell DNA using Zymo Research's EZ DNA Methylation Kit (Irvine, CA, USA) and the manufacturer's instructions, with one modification. Samples were incubated under the Alternative Incubation Conditions for Illumina Infinium Methylation Assay with an increased number of cycles (20 cycles of 95°C for 30 s and 50°C for 15 min) according to the manufacturer's instructions.

### PCR amplification and pyrosequencing

**Figure 1:**
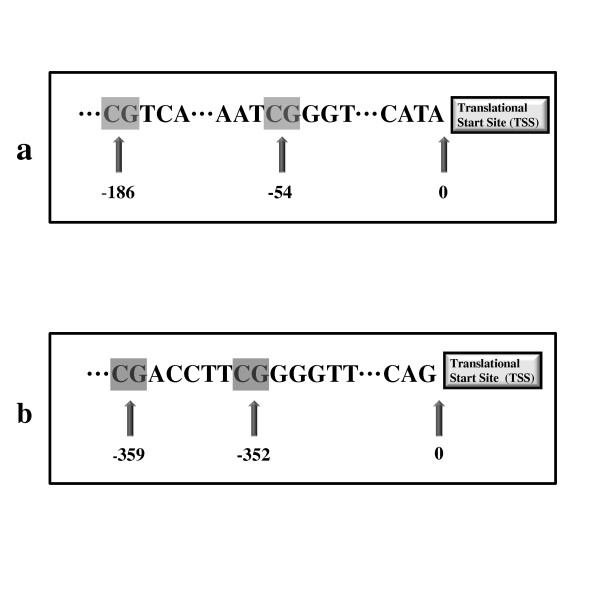
**CpG sites under investigation relative to the translation start site (TSS) for (a) proximal promoter of interferon (IFNγ); (b) proximal promoter of inducible nitric oxide synthase (iNOS)**.

The primers for performing PCR and the PCR product sequencing (Table 1) were designed using PyroMark Assay Design 2.0 software (Qiagen, Valencia, CA) for the regions of interest for IFNγ and iNOS (Figure 1). These targeted areas were chosen based on previous evidence of epigenetic regulation following inhaled environmental exposures [13,23]. PCR reactions were performed with Qiagen HotStarTaq DNA polymerase reagents for IFNγ and iNOS with the following concentrations for each ingredient in the PCR mixtures: 1 × PCR buffer, 1.5 μM of MgCl_2_, 0.2 μM dNTP, 0.4 μM forward primer, 0.4 μM reverse primer. The PCR programs for the IFNγ thermocycler were: 15 min hot start at 95°C, followed by 50 cycles of 95°C for 30 s, 55°C for 1 min, and 72°C for 1 min, with a 10 min elongation at 72°C. The PCR programs for the iNOS thermocycler were: 15 min hot start at 95°C, followed by 50 cycles of 95°C for 30 s, 56°C for 1 min, and 72°C for 1 min 30 s, with a 10 min elongation at 72°C. The PCR product was sequenced using PyroMark Q24 Pyrosequencer after verifying the positive PCR products by visualizing the appropriately sized band on a 1.2% agarose gel. All DNA extractions and bisulfite conversions were performed by the same lab researcher (DZT) who also performed all the IFNγ pyrosequencing. All iNOS pyrosequencing was performed by a second researcher (JSK).

**Table 1:** Primer sequences

Gene	Primer	Sequence
IFNγ	Forward	5'-AGAATGGTATAGGTGGGTATAATGG-3'
	Reverse	5'-Biotin-CAAAACAATATACTACACCTCCTCTA-3'
	Sequencing (CpG^-54^)^a^	5'-ATTATTTTATTTTAAAAAATTTGTG-3'
	Sequencing (CpG^-186^)^a^	5'-GGTGGGTATAATGGGTTTG-3'
iNOS	Forward	5'-TTAGGGTTAGGTAAAGGTATTTTTGTTT-3'
	Reverse	5'-Biotin-CAATTCTATAAAACCACCTAATAATCTTAA-3'
	Sequencing^b^	5'-TAAAGGTATTTTTGTTTTAA-3'

^a^Site based on previous studies of interferon (IFNγ) [15].

^b^Site based on previous studies of inducible nitric oxide synthase (iNOS) [17,23].

### Statistical analysis

Concordance correlations were calculated for all replicate (amplified and sequenced more than one time) and field duplicate (sequential collection of two samples in the field) samples. Within subject correlations were calculated for samples repeated 4 to 7 days later in the same children. For repeat samples, within subject correlations were assessed using SPSS (SPSS, Chicago, Il, USA). Each CpG site was analyzed separately.

## Results

**Figure 2:**
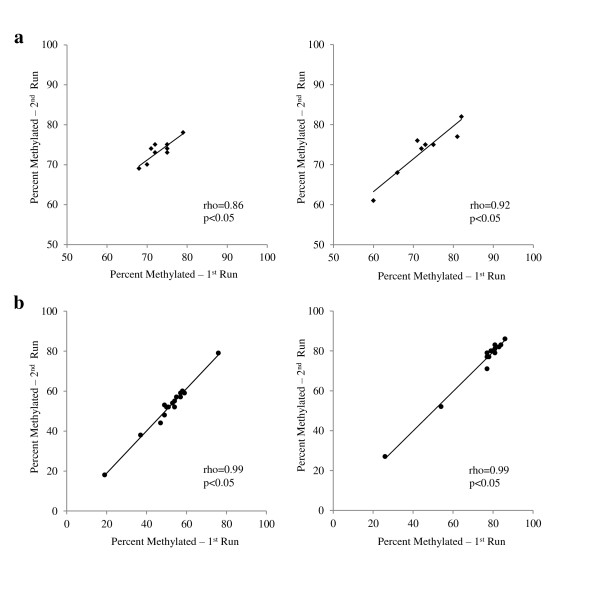
**Concordance correlations between replicate samples for (a) CpG^-186 ^(left, n = 9) and CpG^-54 ^(right, n = 8) on interferon (IFNγ) proximal promoter; (b) CpG^-359 ^(left, n = 16) and CpG^-352 ^(right, n = 14) located in the inducible nitric oxide synthase (iNOS) proximal promoter**. Samples represent repeat pyrosequencing runs of same sample of bisulfite converted DNA.

**Figure 3:**
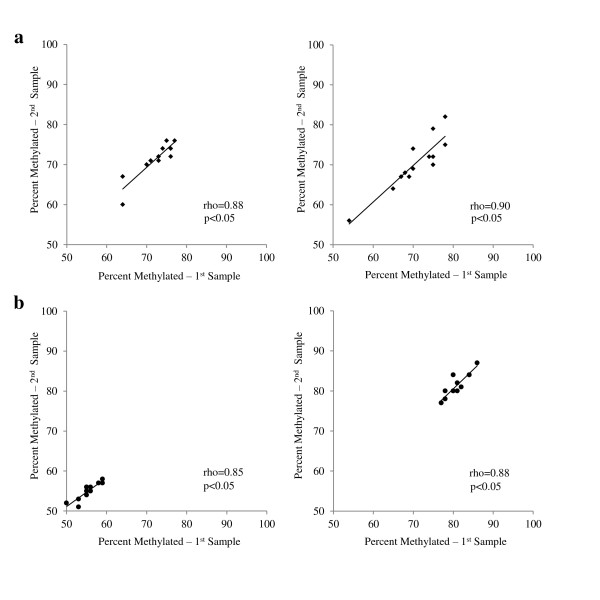
**Concordance correlations between duplicate samples for (a) CpG^-186 ^(left, n = 11) and CpG^-54 ^(right, n = 13) on interferon (IFNγ) proximal promoter; (b) CpG^-359 ^(left, n = 11) and CpG^-352 ^(right, n = 10) located in the inducible nitric oxide synthase (iNOS) proximal promoter proximal promoter**. Samples were collected from sequential buccal swabs minutes apart.

In order to determine whether experimental procedures for PCR and pyrosequencing produced repeatable and consistent data, replicate samples were run. We found that replicate samples were highly correlated for IFNγ and iNOS at all CpG sites tested (Figure 2). For example, the concordance correlations (rho) between the first and second pyrosequencing run for the replicate IFNγ samples were 0.86 (*P *< 0.05) and 0.92 (*P *< 0.05) for CpG^-186 ^and CpG^-54^, respectively; and for iNOS 0.98 (*P *< 0.05) and 0.99 (*P *< 0.05) for CpG^-359 ^and CpG^-352^, respectively. To determine the reproducibility of buccal cell samples collected from children in 'real world' conditions, field duplicate samples were collected. We also found a high correlation between duplicate samples for IFNγ and iNOS at all CpG sites tested (Figure 3). Specifically, the concordance correlations (rho) for IFNγ duplicate samples were 0.88 (*P *< 0.05) and 0.91 (*P *< 0.05) for CpG^-186 ^and CpG^-54^, respectively; and 0.83 (*P *< 0.05) and 0.88 (*P *< 0.05) for iNOS duplicate samples CpG^-359 ^and CpG^-352^, respectively. In addition, the methylation levels of the two iNOS CpG sites correlated highly with each other (Spearman r = 0.77, *P *< 0.05, n = 28), whereas the two IFNγ CpG sites correlated only moderately with each other (r = 0.45, *P *< 0.05, n = 20). Neither iNOS CpG site correlated with either IFNγ site.

**Figure 4:**
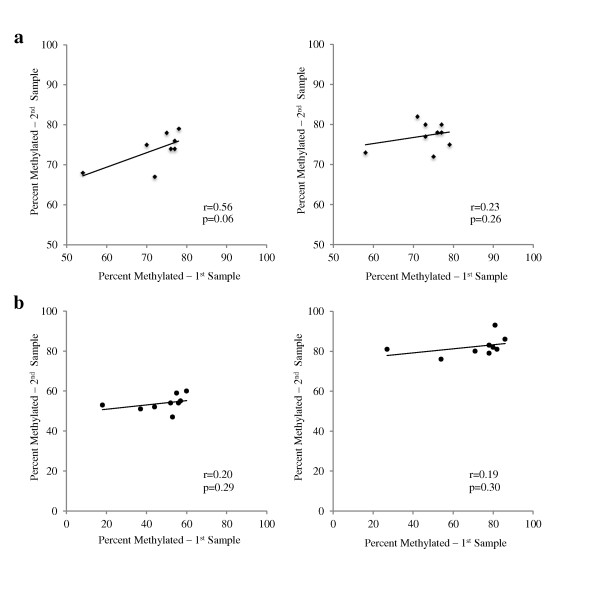
**Within-subject correlations between samples repeated from same subjects 4 to 7 days apart, for (a) CpG^-186 ^(left, n = 8) and CpG^-54 ^(right, n = 9) on the interferon (IFNγ) proximal promoter; (b) CpG^-359 ^(left, n = 9) and CpG^-352 ^(right, n = 9) on the inducible nitric oxide synthase (iNOS) proximal promoter**.

To address a basic question about the time course of DNA methylation, we asked whether buccal cell methylation levels would vary when remeasured days later. We found that repeat samples for iNOS and IFNγ collected 4 to 7 days later demonstrated low within-subject correlations (Figure 4). For example, the within-subject correlations for IFNγ repeat samples were: r = 0.56 (*P *= 0.06) and 0.23 (*P *= 0.26) for CpG^-186 ^and CpG^-54^, respectively; and the within-subject correlations for iNOS repeat samples were: r = 0.20 (*P *= 0.29) and 0.19 (*P *= 0.30) for CpG^-359 ^and CpG^-352^, respectively. These results suggest that DNA methylation levels vary over a 4 to 7 day period within individual subjects.

## Discussion

Our first objective was to assess the reproducibility of buccal DNA collection and quantification of DNA methylation of asthma genes among a cohort of young children. We found that replicate and field duplicate samples correlated strongly. These results suggest our field and laboratory procedures (including bisulfite conversion, pyrosequencing) are robust, and that collection and measure of buccal cell DNA methylation in cohort studies can have great utility. Also they allow us to start answering fundamental questions about the stability of DNA methylation in buccal cells over time, questions that have significant implications for the understanding of environmental epigenetic regulation in children.

Upon confirming the feasibility and reproducibility of these measures, our next step was to assess acute differences in levels of buccal cell DNA methylation of select sites on two asthma genes tested days apart. We found low within-subject correlations for both IFNγ (r = 0.56 and 0.23 for CpG^-186^, CpG^-54^, respectively) and iNOS (r = 0.20 and 0.19 for CpG^-359^, CpG^-352^, respectively) upon repeat testing over a 4 to 7 day period. Combined with the high level of reproducibility previously demonstrated, these data suggest that methylation levels can change acutely in both genes. These findings are novel in that research measuring short-term changes in methylation has been limited, with only a few examples to date [23,26]. For example, Baccarelli and colleagues tested blood DNA methylation levels in long interspersed nucleotide elements (LINE 1) and Alu element (Alu) as surrogates of global methylation levels after acute exposure to black carbon/soot. They found an association between ambient black carbon levels and LINE 1, but not Alu, demethylation suggesting that global epigenetic regulation may occur in association with measures of recent air pollution exposure. As a second example, Tarantini and colleagues, in addition to assessing acute changes in global methylation (LINE 1, Alu), assessed PBMC DNA methylation levels in the proinflammatory iNOS gene over a 3-day period. Interestingly, they found an association between concentrations of PM_10 _particles and iNOS demethylation, implicating this regulator of airway inflammation as a gene whose expression may depend in part on alteration of DNA methylation levels.

We reasoned that genes expressed in buccal cells, like those in peripheral blood mononuclear cells, also could undergo acute changes in DNA methylation, presumably following recent changes in environmental triggers. Inducible NOS was a main focus of this investigation because of Tarantini's and colleagues' reports, and because of its reported expression in the buccal mucosa [27]. Interestingly, methylation levels and interquartile ranges observed in this study of inner city asthmatic children were strikingly similar to the report by Breton and colleagues in a Southern California cohort, suggesting that some of our new results may be generalizable to other cohorts.

The second main focus, namely susceptibility of buccal cells to undergo DNA methylation in the promoter region of IFNγ, was in response to considerable previous work that suggests methylation of IFNγ is critical to its gene expression [15,28]. This body of work also includes our previous findings in mice that CD4+ T cells undergo increases in IFNγ DNA methylation in multiple CpG sites following exposures to diesel exhaust particles. In these experiments, methylation levels were measured once after 3 weeks of diesel exposure. The hypermethylation that occurred over this period was suspected to induce silencing of the IFNγ gene and downregulation of the production of proallergic IgE antibodies, as indicated by the observed inverse correlation between IFNγ methylation levels and IgE [13]. Indeed the CpG sites studied here (CpG^-186 ^and CpG^-54^) are conserved in mice [28]. One of the few studies of human cells to date, conducted by Gonsky and colleagues, looked at the same loci in the promoter of IFNγ (CpG^-186 ^and CpG^-54^) in lamina propria T cells and peripheral blood T cells [29]. Their group found that a 5% reduction in methylation of CpG^-54 ^in the promoter region of IFNγ was associated with a threefold increase in IFNγ gene expression. While our study did not link the changes in IFNγ promoter methylation in buccal cells over time with downstream biological events as the Liu *et al*. and Gonsky *et al*. studies did, it does for the first time show that the time course for changes in DNA methylation can be as short as several days in a pediatric cohort.

We acknowledge several limitations to the study. First, a limited number of asthma genes, and CpG sites per gene, were studied. Examination of additional CpG sites and asthma genes may help elucidate the time course of epigenetic change of other genes important to airway inflammation. To date it still needs to be ascertained how methylation levels across multiple CpG sites may impact gene transcription differentially, though early evidence suggests that particular sites, such as in proximal gene promoters such as IFNγ CpG^-54^, may be critical [28]. Alternately, other evidence suggests that CpG methylation in the intron could affect elongation and thereby gene transcription [30,31]. Also, the sample size was small, though sufficient to evaluate the quality of the reproduced data. The data display ranges of methylation that may be shown to be biologically meaningful in future studies. The buccal cell collection does not test for cell specific effects nor necessarily represent what occurs in respiratory epithelium. The magnitude of changes may vary across tissues. Moreover, in the absence of corresponding gene expression data, it may be difficult to know whether their epigenetic changes led to downstream molecular events. Repeat findings in other cohorts would be helpful to validate these results.

## Conclusions

In summary, these findings suggest that buccal sampling is a feasible, non-invasive technique that yields reproducible results. The low correlations found during repeat sampling, especially when contrasted with highly correlated replicate and duplicate samples, suggest that changes in the level of DNA methylation can occur acutely, over a 4 to 7 day period. Given the dynamism of these epigenetic marks, one could speculate that these epigenetic marks are responsive to the rapidly changing environmental exposures. Asthma is a complex environmentally related disease with a rising US childhood prevalence of 9.4% [32], reaching as high as 28.5% in some areas of New York City [33]. By 2025, asthma is estimated to affect over 100 million people worldwide [34]. Understanding the triggers for asthma exacerbations and their associated molecular immune responses requires longitudinal studies that carefully pair environmental measures with relevant epigenetic biomarkers and clinical outcomes. These results suggest that such work can be conducted safely and accurately in an inner city pediatric cohort through buccal cell sampling and pyrosequencing of asthma genes.

## Competing interests

The authors declare that they have no competing interests.

## Authors' contributions

DZT participated in the design and coordination of the study, and drafted the manuscript. JSK participated in the conduction and coordination of the study, its statistical analysis and edited the manuscript. KM participated in the conduction of the study, and edited the manuscript. HJ participated in the conduction of the study and edited the manuscript. MMN participated in the conduction of the study and edited the manuscript. FPP participated in the study design and edited the manuscript. RLM designed and coordinated the study, participated in the statistical analysis, and drafted the manuscript. All authors read and approved the final manuscript.

## References

[B1] Patel MM Hoepner L Garfinkel R Chillrud S Reyes A Quinn JW Perera F Miller RL Ambient metals, elemental carbon, and wheeze and cough in New York City children through 24 months of age Am J Respir Crit Care Med 2009 180 1107 1113 10.1164/rccm.200901-0122OC 19745205 PMC2784415

[B2] Patel MM Miller RL Air pollution and childhood asthma: recent advances and future directions Curr Opin Pediatr 2009 21 235 242 10.1097/MOP.0b013e3283267726 19663041 PMC2740858

[B3] Romieu I Barraza-Villarreal A Escamilla-Nun C Almstrand AC Diaz-Sanchez D Sly PD Olin AC Exhaled breath malondialdehyde as a marker of effect of exposure to air pollution in children with asthma J Allergy Clin Immunol 2008 121 903 909 10.1016/j.jaci.2007.12.004 18234317

[B4] Gilliland FD McConnell R Peters J Gong H Jr A theoretical basis for investigating ambient air pollution and children's respiratory health Environ Health Perspect 1999 107 Suppl 3 403 407 10.1289/ehp.99107s3403 10346989 PMC1566227

[B5] Liu L Poon R Chen L Frescura AM Montuschi P Ciabattoni G Wheeler A Dales R Acute effects of air pollution on pulmonary function, airway inflammation, and oxidative stress in asthmatic children Environ Health Perspect 2009 117 668 674 10.1289/ehp11813 19440509 PMC2679614

[B6] Riedel M Diaz-Sanchez D Biology of diesel exhaust effects on respiratory function J Allergy Clin Immunol 2005 115 221 228 10.1016/j.jaci.2004.11.047 15696072

[B7] Diaz-Sanchez D Garcia MP Wang M Jyrala M Saxon A Nasal challenge with diesel exhaust particles can induce sensitization to a neoallergen in the human mucosa J Allergy Clin Immunol 1999 104 1183 1188 10.1016/S0091-6749(99)70011-4 10588999

[B8] Diaz-Sanchez D Proietti L Polosa R Diesel fumes and the rising prevalence of atopy: an urban legend? Curr Allergy Asthma Rep 2003 3 146 152 10.1007/s11882-003-0027-4 12562554

[B9] Bateson TF Schwartz J Children's response to air pollutants J Toxicol Environ Health A 2008 71 238 243 18097949 10.1080/15287390701598234

[B10] Miller RL Ho SM Environmental epigenetics and asthma: current concepts and call for studies Am J Respir Crit Care Med 2008 177 567 573 10.1164/rccm.200710-1511PP 18187692 PMC2267336

[B11] Kuriakose JS Miller RL Environmental epigenetics and allergic diseases: recent advances Clin Exp Allergy 2010 40 1602 1610 10.1111/j.1365-2222.2010.03599.x 20718778 PMC2970703

[B12] Perera F Tang WY Herbstman J Tang D Levin L Miller R Ho SM Relation of DNA methylation of 5'-CpG island of ACSL3 to transplacental exposure to airborne polycyclic aromatic hydrocarbons and childhood asthma PLoS ONE 2009 4 e4488 10.1371/journal.pone.0004488 19221603 PMC2637989

[B13] Liu J Ballaney M Al-alem U Quan C Jin X Perera F Chen LC Miller RL Combined inhaled diesel exhaust particles and allergen exposure alter methylation of T helper genes and IgE production *in vivo* Toxicol Sci 2008 102 76 81 18042818 10.1093/toxsci/kfm290 PMC2268643

[B14] Cao D Bromberg PA Samet JM COX-2 expression induced by diesel particles involves chromatin modification and degradation of HDAC1 Am J Respir Cell Mol Biol 2007 37 232 239 10.1165/rcmb.2006-0449OC 17395887

[B15] White GP Hollams EM Yerkovich ST Bosco A Holt BJ Bassami MR Kusel M Sly PD Holt PG CpG methylation patterns in IFNγ promoter in naive T cells: variations during Th1 and Th2 differentiation and between atopics and non-atopics Pediatr Allergy Immu 2006 17 557 564 10.1111/j.1399-3038.2006.00465.x 17121582

[B16] Kwon NH Kim JS Lee JY Oh MJ Choi DC DNA methylation and the expression of IL-4 and IFN-γ promoter genes in patients with bronchial asthma J Clin Immunol 2008 28 139 146 10.1007/s10875-007-9148-1 18004650

[B17] Breton CV Byun HM Wang X Salam MT Siegmund K Gilliland FD DNA methylation in the ARG-NOS pathway is associated with exhaled nitric oxide in asthmatic children Am J Respir Crit Care Med 2011 184 191 197 10.1164/rccm.201012-2029OC 21512169 PMC3172885

[B18] Sridhar S Schembri F Zeskind J Shah V Gustafson AM Steiling K Liu G Dumas YM Zhang X Brody JS Lenburg ME Spira A Smoking-induced gene expression changes in the bronchial airways are reflected in nasal and buccal epithelium BMC Genom 2008 9 259 10.1186/1471-2164-9-259 PMC2435556 18513428

[B19] Johanning GL Heimburger DC Piyathilake CJ DNA methylation and diet in cancer J Nutrition 2002 132 3814S 3818S 12468630 10.1093/jn/132.12.3814S

[B20] Bhutani M Pathak AK Fan YH Liu DD Lee JJ Tang H Kurie JM Morice RC Kim ES Hong WK Mao L Oral epithelium as a surrogate tissue for assessing smoking-induced molecular alterations in the lungs Cancer Prev Res 2009 1 39 44 10.1158/1940-6207.CAPR-08-0058 PMC4183362 19138934

[B21] Moseley PL Hemken C Monick MM Nugent KM Hunninghake GW Interferon and growth factor activity for human lung fibroblasts: release from bronchoalveolar cells from patients with active sarcoidosis Chest 1986 89 657 662 10.1378/chest.89.5.657 3084179

[B22] Schoenborn JR Dorschner M Sekimata M Santer DM Shnyreva M Fitzpatrick DR Stamatoyannopoulos JA Wilson CB Comprehensive epigenetic profiling identifies multiple distal regulatory elements directing *IFN*-γ transcription Nat Immunol 2007 8 732 742 17546033 10.1038/ni1474 PMC2144744

[B23] Tarantini L Bonzini M Apostoli P Pegoraro V Bollati V Marinelli B Cantone L Rizzo G Hou L Schwartz J Bertazzi PA Baccarelli A Effects of particulate matter on genomic DNA methylation content and iNOS promoter methylation Environ Health Perspect 2009 117 217 222 19270791 10.1289/ehp.11898 PMC2649223

[B24] Perera FP Rauh V Tsai WY Kinney P Camann D Barr D Bernert T Garfinkel R Tu YH Diaz D Dietrich J Whyatt RM Effects of transplacental exposure to environmental pollutants on birth outcomes in a multiethnic population Environ Health Perspect 2003 111 201 205 12573906 10.1289/ehp.5742 PMC1241351

[B25] Whyatt RM Barr DB Camann DE Kinney PL Barr JR Andrews HF Hoepner LA Garfinkel R Hazi Y Reyes A Ramirez J Cosme Y Perera FP Contemporary-use pesticides in personal air samples during pregnancy and blood samples at delivery among urban minority mothers and newborns Environ Health Perspect 2003 111 749 756 12727605 10.1289/ehp.5768 PMC1241486

[B26] Baccarelli A Wright RO Bollati V Tarantini L Litonjua AA Suh HH Zanobetti A Sparrow D Vokonas PS Schwartz J Rapid DNA methylation changes after exposure to traffic particles Am J Respir Crit Care Med 2009 179 572 578 10.1164/rccm.200807-1097OC 19136372 PMC2720123

[B27] Arany I Brysk MM Brysk H Tyring SK Regulation of inducible nitric oxide synthase mRNA levels by differentiation and cytokines in human keratinocytes Biochem Biophys Res Commun 1996 220 618 622 10.1006/bbrc.1996.0452 8607813

[B28] Jones B Chen J Inhibition of IFN-γ transcription by site-specific methylation during T helper cell development EMBO J 2006 25 2443 2452 10.1038/sj.emboj.7601148 16724115 PMC1478170

[B29] Gonsky R Deem RL Targan SR Distinct methylation of IFNγ in the gut J Interferon Cytokine Res 2009 29 407 414 10.1089/jir.2008.0109 19450149 PMC2956574

[B30] Lorincz MC Dickerson DR Schmitt M Groudine M Intragenic DNA methylation alters chromatin structure and elongation efficiency in mammalian cells Nat Struct Mol Biol 2004 11 1068 1075 10.1038/nsmb840 15467727

[B31] Klose RJ Bird AP Genomic DNA methylation: the mark and its mediators Trends Biochem Sci 2006 31 89 97 10.1016/j.tibs.2005.12.008 16403636

[B32] Centers for Disease Control and Prevention Asthma Factsheet http://www.cdc.gov/nchs/fastats/asthma.htm

[B33] Nicholas SW Jean-Louis B Ortiz B Northridge M Shoemaker K Vaughan R Rome M Canada G Hutchinson V Addressing the childhood asthma crisis in Harlem: the Harlem Children's Zone Asthma Initiative Am J Public Health 2005 95 245 249 10.2105/AJPH.2004.042705 15671459 PMC1449161

[B34] World Health Organization Global surveillance, prevention and control of chronic respiratory diseases: a comprehensive approach http://www.who.int/gard/publications/GARD Book 2007.pdf

